# The Treatment of Fever Following Delivery.—Serum Therapy in Puerperal Fever

**Published:** 1899-11-04

**Authors:** 


### THE TREATMENT OF FEVER FOLLOWING DELIVERY.—SERUM THERAPY IN PUERPERAL FEVER.

Speaking at Portsmouth upon the use of serum therapy in the treatment of fever following delivery, Dr. Herbert Spencer defined puerperal fever as "fever during the puerperium caused by microbic infection from without." Pyrexia during the puerperium might, he said, be set up by many causes. (1) It might be what is called "one day fever," arising about the third day, possibly set up by copræmia, and commonly relieved by the mild aperient usually given on that day. (2) Fever might be caused by complications not referable to the labour, such as influenza, acute phthisis, and typhoid fever. (3) Fever might be caused by internal infection arising from pre-existing lesions, such as ovarian or fibroid tumours, stone in the bladder, pyosalpinx, &c. (4) Fever might be set up by external infection, and this was what we understood by the term puerperal fever. Till lately this disease was thought to be due to the streptococcus pyogenes, but recent bacteriological investigation has shown that the micro-organisms associated with puerperal fever are very varied in character, including as well as the above, staphylococci, colon bacilli, gonococci, anaërobic bacilli, diphtheria bacilli, and gas and typhoid bacilli. In regard to treatment prophylaxis is of the greatest importance—careful examination towards the end of pregnancy, avoidance of injury during labour, careful management of the third stage, and thorough disinfection of the vulva and of the surgeon's hands and instruments. When puerperal fever has arisen it is necessary in the first place that a thorough examination of the patient and the uterus be made, for in many cases, apparently almost hopeless, putrid portions of placenta may be found in the uterus, and their removal may be followed by the recovery of the patient. Again, it may be possible, as the result of such an examination, to enucleate a fibroid tumour, changes in which may be at the root of the mischief. In emptying the uterus the finger should be employed, sometimes large blunt-ended forceps, but never the curette after labour at full term, for the uterus may be so soft that the danger of perforation may be a very real one indeed. Passing over other methods and proceedings which may be found necessary in the treatment of puerperal fever, we come to the use of serum. *A priori* it would not appear to be a scientific treatment to administer an antitoxin produced by the streptococcus in the treatment of a disease which is by no means generally associated with that micro-organism. This does not mean that it might not be beneficial in cases due to the streptococcus; but even in regard to this the experience of the committee of the American Gynæcological Society showed that the mortality was 33 per cent. after its use in cases in which the streptococci were positively demonstrated, and it is doubtful whether the natural mortality is as much as this. The conclusions, then, at which Professor Herbert Spencer arrives are: (1) That as usually applied it has not a scientific basis; (2) that it has not lowered the mortality of puerperal sepsis; (3) that it usually lowers the temperature, and sometimes improves the general condition; and (4) that its use is not free from danger. Whether it will prove of value in the treatment of pure streptococcic infection, as detected by examination of the uterus or blood, remains for the future to show.

